# Exploring the untapped catalytic application of a ZnO/CuI/PPy nanocomposite for the green synthesis of biologically active 2,4,5-trisubstituted imidazole scaffolds†

**DOI:** 10.1039/d3na00077j

**Published:** 2023-03-20

**Authors:** Sahil Kohli, Nisha, Garima Rathee, Sunita Hooda, Ramesh Chandra

**Affiliations:** a Drug Discovery & Development Laboratory, Department of Chemistry, University of Delhi Delhi-110007 India rameshchandragroup@gmail.com; b Department of Chemistry, Acharya Narendra Dev College, University of Delhi Delhi-110019 India hooda_sunita@hotmail.com; c Dr. B.R. Ambedkar Center for Biomedical Research (ACBR), University of Delhi Delhi-110007 India; d Institute of Nanomedical Science (INMS), University of Delhi Delhi-110007 India

## Abstract

This work is focused on designing an innovative, efficient, and reusable heterogeneous ZnO/CuI/PPy nanocomposite *via* the self-assembly approach where pyrrole is oxidized into polypyrrole (PPy) and pyrrole also behaves as a reductant in the presence of KI. This so-obtained material was characterized by XRD, FTIR, FESEM, EDX, TEM, XPS, and ICP. TEM clearly shows a spherical morphology with the particle size ranging between 18 and 42 nm. The fabricated nanomaterial was tested for one-pot catalytic synthesis of biologically active 2,4,5-trisubstituted imidazoles under solvent-free conditions. The present work includes the benefits of an easy work-up procedure, higher product yield, shorter reaction duration, and no additional additive requirement under green and sustainable conditions. Moreover, the catalyst exhibited reusability for six runs with no considerable reduction in the respective yields and reactivity (confirmed by XRD, SEM, and TEM of the recycled catalyst). The ICP study shows very low leaching of copper (2.08 ppm) and zinc (0.12 ppm) metals. The approach also presented better values of green metrics like the E-factor, process mass intensity, carbon efficiency and reaction mass efficiency.

## Abstract 2

The fabricated catalyst ZnO/CuI/PPy facilitates the synthesis of 2,4,5-trisubstituted imidazole under solvent free conditions with ideal values of green metrics.

## Graphical abstract

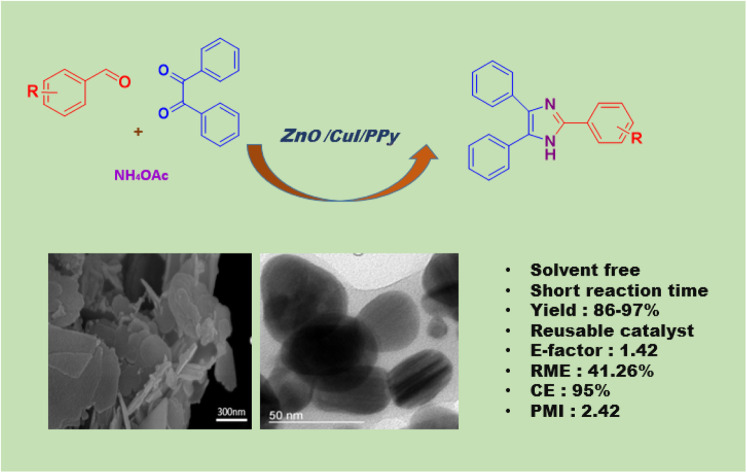

## 1 Introduction

Multicomponent reactions (MCRs) have emerged as a potent tool for rapidly and efficiently constructing interesting biologically active compounds.^1^ Reactants such as aldehydes/ketones, isonitriles, ammonia/amines, carboxylic acids and their derivatives are commonly used for multicomponent reactions. These reactions offer significant benefits over conventional syntheses, such as simple procedures, waste prevention, high atom economy, and convergent character.^2–4^

**Fig. 1:**
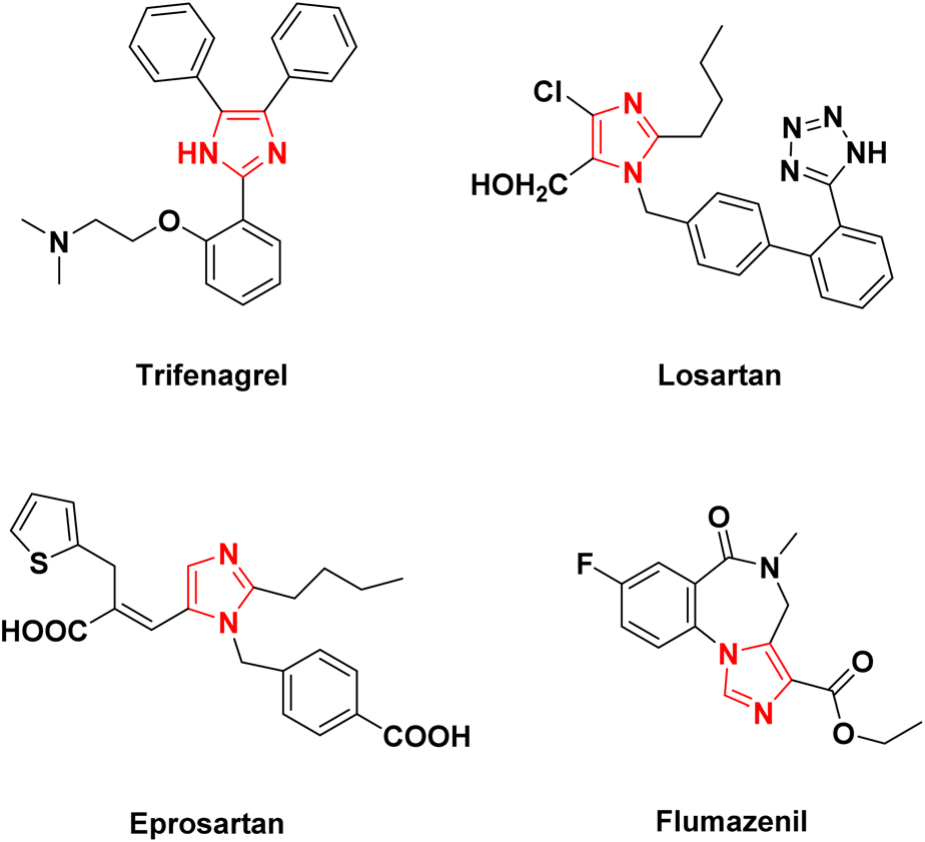
Drugs containing imidazole scaffolds.

Imidazoles are a significant category of heterocyclic compounds that have gained attention based on their numerous biological and pharmacological actions such as anti-inflammatory properties,^5^ and as glucagon receptor antagonists,^6^ antitumor agents,^7^ antiulcerative agents,^8^ proton pump inhibitors,^9^ and pesticides.^10^ Some of the biological imidazole containing drugs are shown in Fig. 1. Moreover, the substituted imidazoles have additional applications such as organocatalysts in metalloenzymes, functional materials in organic electroluminescent devices, precursors for stable carbene ligands, conjugated and functional polymers, and ionic liquids.^11^ Various catalysts have been used in synthesizing substituted imidazole such as acetic acid,^12^ silica sulfuric acid,^13^ ZrCl_4_,^14^ InCl_3_·3H_2_O,^15^l-proline,^16^ I_2_,^17^ and DABCO.^18^ However these methods encountered several drawbacks like the use of toxic and expensive reagents, harsh reaction conditions, prolonged reaction times and low product yields. As a result, there is a broad scope for developing efficient, clean, and environmentally benign methods for synthesizing such compounds.

Nanomaterials seem to be sustainable substitutes to other conventional materials as catalyst supports, robust and heterogeneous catalysts with high surface area. The small nano-sized particles enhance the exposed surface area of the active part of the catalyst. Also, selectivity and activity of nanocatalysts can be controlled by modifying physical and chemical properties such as the shape, size, morphology and metal support.^19^ Among various inorganic metal oxides, zinc oxide (ZnO) is an essential material in research due to its widespread applications and unique properties. ZnO is a non-toxic metal oxide that low-cost methods can synthesize. It has been discovered to be an effective catalyst for several organic transformations due to numerous environmental benefits such as minimum waste production and execution time, accessible transport and catalyst recycling.^20^

Polypyrrole (PPy) is one of the most promising polymers because of its facile synthesis, high conductivity, good environmental stability, and usage as a support of noble metals, namely, Cu and Pd, for application in heterogeneous catalysis.^21^ Among various noble metals, copper is a very soft and malleable element with high thermal and electrical conductivity. Due to its environmentally benign nature, high natural abundance, low cost and unique properties of showing various oxidation states, such as Cu(0, I, II, III), Cu-based nanocatalysts have discovered numerous applications in nanotechnology, including electrocatalysis, photocatalysis and organic catalytic transformations.^22–24^ Among copper nanoparticles, cuprous iodide (CuI) has attracted much interest due to its several applications as an adsorbent for heavy metal pollutants and nanocatalysts for organic transformations.^25^

In continuation of our work towards fabricating various nanocatalysts for the greener synthesis of numerous biologically active scaffolds.^26–29^ herein, we design a ZnO/CuI/PPy nanocatalyst to synthesize 2,4,5-trisubstituted imidazole using benzil, aldehyde, and ammonium acetate under neat conditions. The nanocomposite was characterized using FTIR, XRD, EDX, FESEM, TEM, XPS and ICP. The material was stable under the performed reaction conditions, resulting in excellent product yield. Also, it was recovered quickly and recycled for upto six cycles without much decrease in % yield.

## 2 Results and discussion

### 2.1 Design and synthesis of the nanocatalyst

**Fig. 2:**
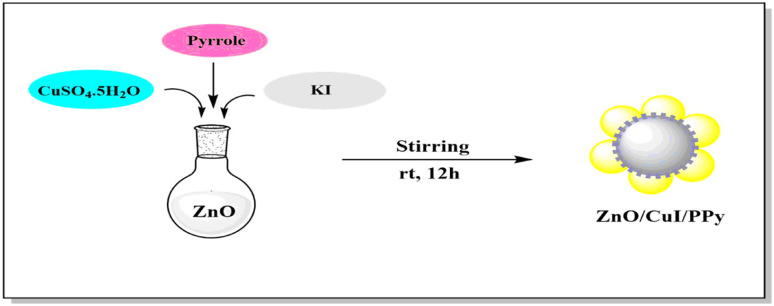
Schematic diagram for the fabrication of the ZnO/CuI/PPy nanocatalyst.

The design and synthesis of the catalyst are illustrated in Fig. 2. It involves the synthesis of the catalyst by a simple one pot method which includes stirring for 12 hours at room temperature resulting in the ZnO/CuI/PPy nanocatalyst.

**Fig. 3:**
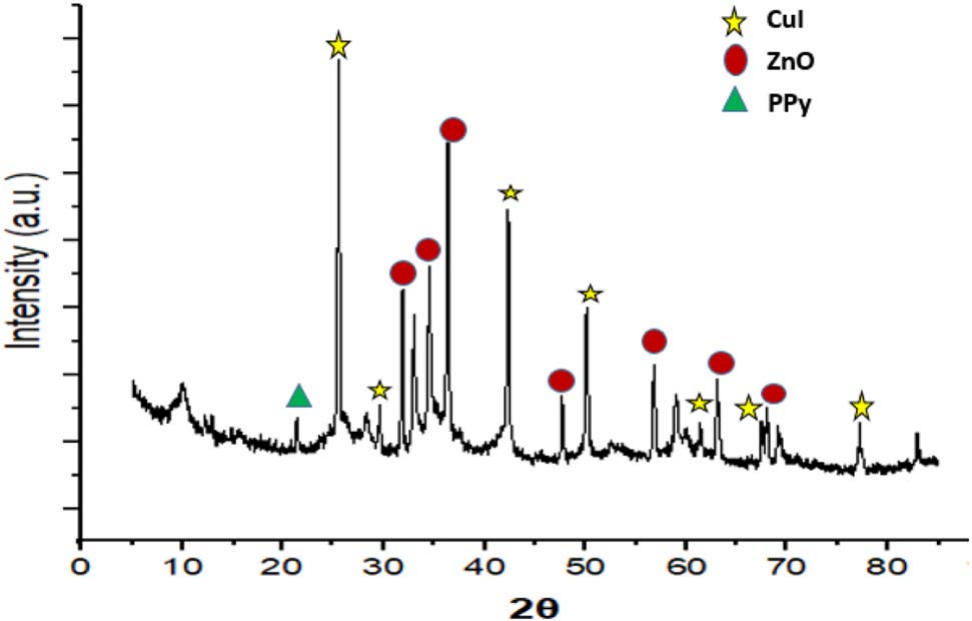
XRD spectrum of the ZnO/CuI/PPy nanocatalyst.

Fig. 3 shows the XRD spectrum of the ZnO/CuI/PPy nanocatalyst. The peaks at the values of 2*θ* = 32.0, 34.5, 36.4, 47.6, 56.7, 62.5 and 68.8° corresponding to (100), (002), (101), (102), (110), (103) and (201) are in agreement with regular patterns of ZnO, whereas the peaks at 2*θ* = 25.6, 29.6, 25.1, 32.8, 39.6, 43.2 and 47.4° corresponding to (111), (200), (220), (311), (400), (331) and (422) of CuI are also in agreement with the JCPDF file (06-0246).^30,31^ The peak at 2*θ* around 20° represents the presence of polypyrrole.^32^

**Fig. 4:**
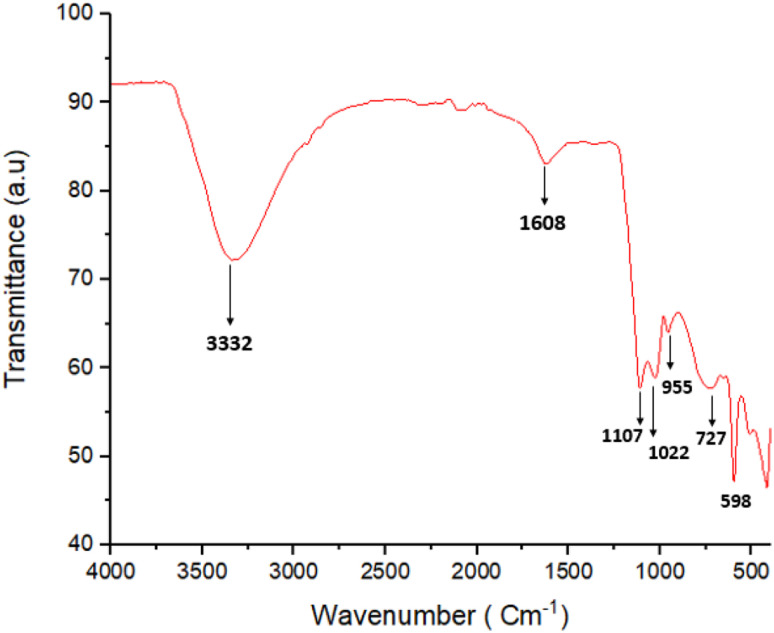
FTIR spectrum of the ZnO/CuI/PPy nanocatalyst.

Fig. 4 shows the FT-IR spectrum obtained for the synthesized ZnO/CuI/PPy nanocatalyst. The characteristic peak for the fundamental vibration of PPy was observed at 1608 cm^−1^.^33^ The peak at 1107 cm^−1^ could be attributed to the vibration peak of C–N.^34^ Also, the vibration peak of C=C was observed at 1022 cm^−1^, and for C–H vibration of the five-membered ring of PPy, a peak was observed at 955 cm^−1^.^35^ These peaks confirmed the presence of polypyrrole. The broad band at 3332 cm^−1^ could be allotted to O–H stretching vibrations of ZnO. Moreover, the peaks at 727 and 598 cm^−1^ are related to the symmetric and asymmetric stretching of the zinc hydroxyl groups, respectively.^36^

**Fig. 5:**
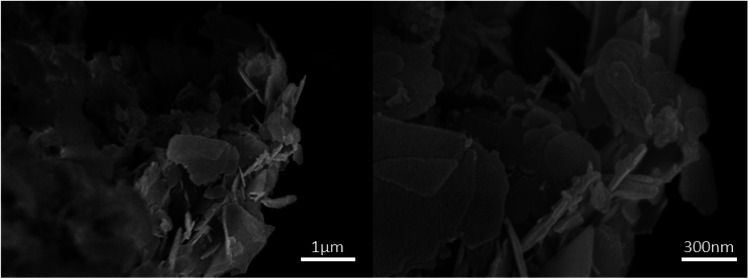
FESEM of the ZnO/CuI/PPy nanocatalyst.

**Fig. 6:**
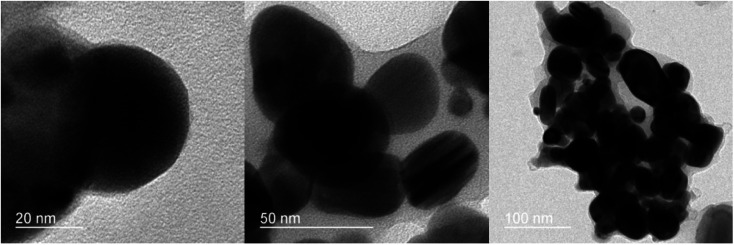
TEM of the ZnO/CuI/PPy nanocatalyst.

The morphology of the fabricated nanocomposite was characterized by FESEM (surface morphology) and TEM (internal morphology) techniques as illustrated in Fig. 5 and 6. The FESEM images depict the smooth flake-like morphology. The TEM images at 20, 50 and 100 nm shows discreet spherical particles in the size range of 18–42 nm.

**Fig. 7:**
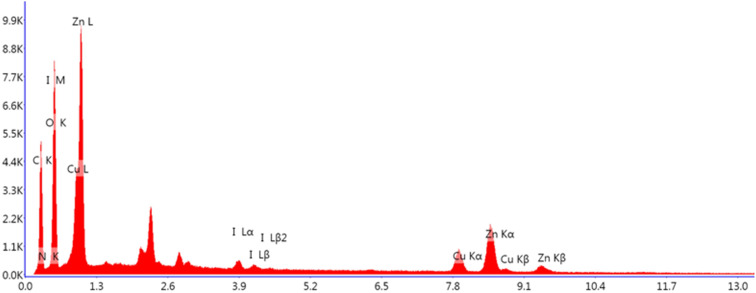
EDAX of the ZnO/CuI/PPy nanocatalyst.

The existence of copper (7.3 wt%), iodine (1.56 wt%), oxygen (33.12 wt%), zinc (21.13 wt%), carbon (27.79 wt%) and nitrogen (9.11 wt%) in the ZnO/CuI/PPy nanocatalyst was examined by energy-dispersive X-ray analysis (EDAX) as shown in Fig. 7.

**Fig. 8:**
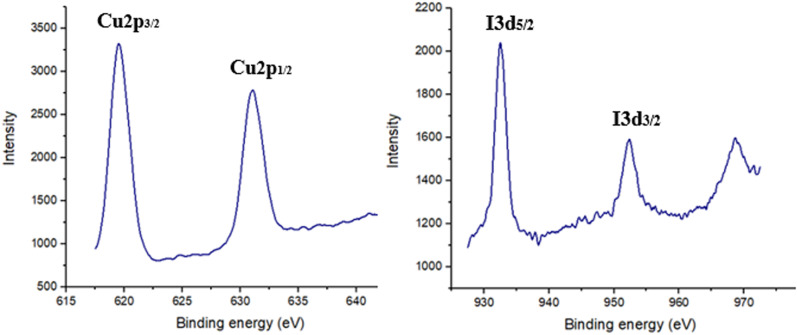
XPS spectrum in the region of Cu 2p and I 3d of the ZnO/CuI/PPy nanocatalyst.

Furthermore, the existence of CuI in the ZnO/CuI/PPy nanocatalyst was confirmed by X-ray photoelectron spectroscopy as displayed in Fig. 8. The values at 619 and 632 eV were associated with I 3p and values at 932.4 and 952.3 eV were associated with Cu 2p. The values were found to be similar to the existing data of CuI nanoparticles that confirmed the +1 oxidation state of copper.^37^

#### 2.1.1 Catalytic activity of the ZnO/CuI/PPy nanocatalyst

##### 2.1.1.1 ZnO/CuI/PPy as a nanocatalyst for the synthesis of imidazole scaffolds

To know the suitable conditions for the reaction and to obtain high % yields of 2,4,5-trisubstituted imidazoles, various parameters have been optimized by using 4-methylbenzaldehyde, benzil and ammonium acetate as the model reaction partners. When no catalyst and solvent were used, the product was formed in 51% (entry 1, Table 1). When solvents such as DMF and DMSO, which are polar aprotic, were used, it was found that the product formed was in trace amounts in both DMF and DMSO (entries 2–3, Table 1). When solvents like ethanol, methanol, water, and EG (ethylene glycol) were used, no reaction in ethanol, methanol and water was observed, but in EG, the product formed was 88% (entries 4–7, Table 1). When the reaction was performed under neat conditions, the product formed was 95% (entry 8, Table 1). Then when the catalyst amount was decreased to 10 mg, yield was 77%, and when increased to 30 mg, yield was 95% (entries 9–10, Table 1). Next, the influence of temperature was studied, and the yield was found to be less when the temperature was decreased to 80 °C and it remained the same when the temperature was increased to 120 °C (entries 11–12, Table 1). Moreover, the reaction progress was determined after 20 and 30 minutes and the yield was found to be 61 and 83%, respectively (entries 13–14, Table 1). Hence, the appropriate reaction condition was 20 mg of ZnO/CuI/PPy for 40 min under solvent-free conditions.

**Table 1:** ZnO/CuI/PPy nanocatalyst used for optimization in the synthesis of 2,4,5-trisubstituted imidazole by using benzil (1), *p*-methylbenzaldehyde (2) and ammonium acetate (3)^a^

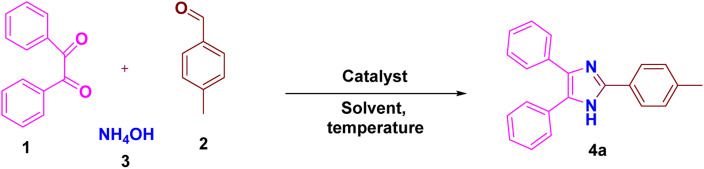					
Entry	Catalyst/amount (mg)	Solvent	Temp. (°C)	Time (min)	Yield (%)
1	—	—	100	40	51
2	ZnO/CuI/PPy (20)	DMF	100	40	Trace
3	ZnO/CuI/PPy (20)	DMSO	100	40	Trace
4	ZnO/CuI/PPy (20)	Ethanol	Reflux	40	—
5	ZnO/CuI/PPy (20)	Methanol	Reflux	40	—
6	ZnO/CuI/PPy (20)	Water	100	40	—
7	ZnO/CuI/PPy (20)	EG	100	40	88
**8**	**ZnO/CuI/PPy (20)**	**Neat**	**100**	**40**	**95**
9	ZnO/CuI/PPy (10)	Neat	100	40	77
10	ZnO/CuI/PPy (30)	Neat	100	40	95
11	ZnO/CuI/PPy (20)	Neat	80	40	82
12	ZnO/CuI/PPy (20)	Neat	120	40	95
13	ZnO/CuI/PPy (20)	Neat	100	20	61
14	ZnO/CuI/PPy (20)	Neat	100	30	83

a Reaction conditions: benzil 1 (1 mmol), aldehyde 2 (1 mmol), ammonium acetate 3 (5 mmol), ZnO/CuI/PPy (10–30 mg) and solvent (4 mL) were stirred at the mentioned temperature.

Derivatives of 2,4,5-trisubstituted imidazole were synthesized using the most appropriate conditions as shown in Table 2. As a result, excellent yields of the products were obtained in all cases from the benzaldehyde precursors (4a–4l).

**Table 2:** Green metrics values

Entry	Green metrics	Ideal values^39^	Present method
1	Process mass intensity	1	2.42
2	E factor	0	1.42
3	Reaction mass efficiency	100%	41.26%
4	Carbon efficiency	100%	95%

**Fig. 9:**
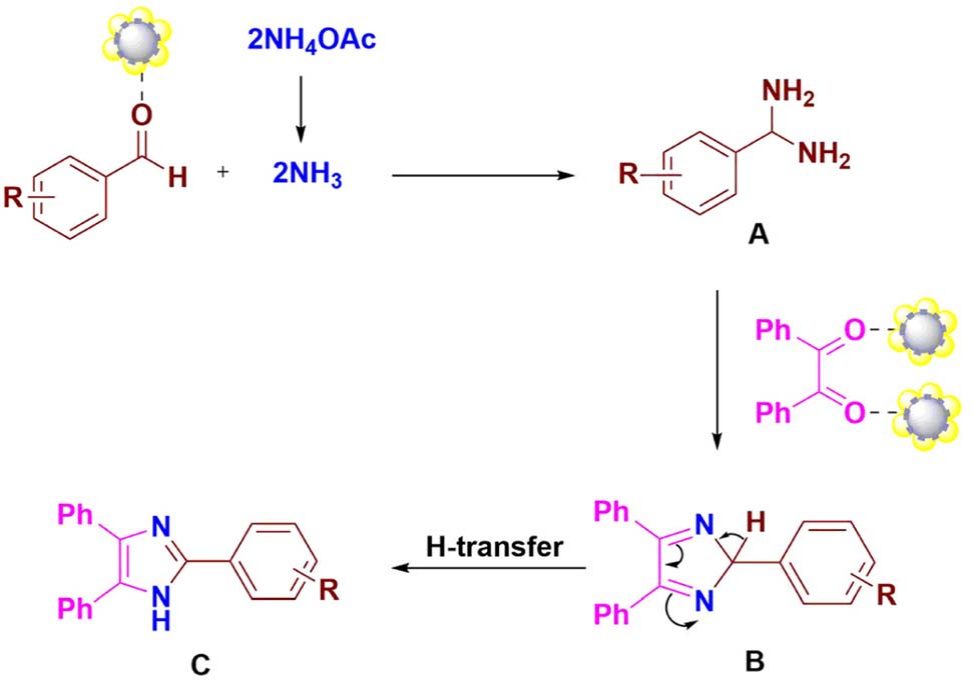
Possible mechanism for synthesis of 2,4,5-trisubstituted imidazole by using the ZnO/CuI/PPy nanocatalyst.

**Scheme 1:**
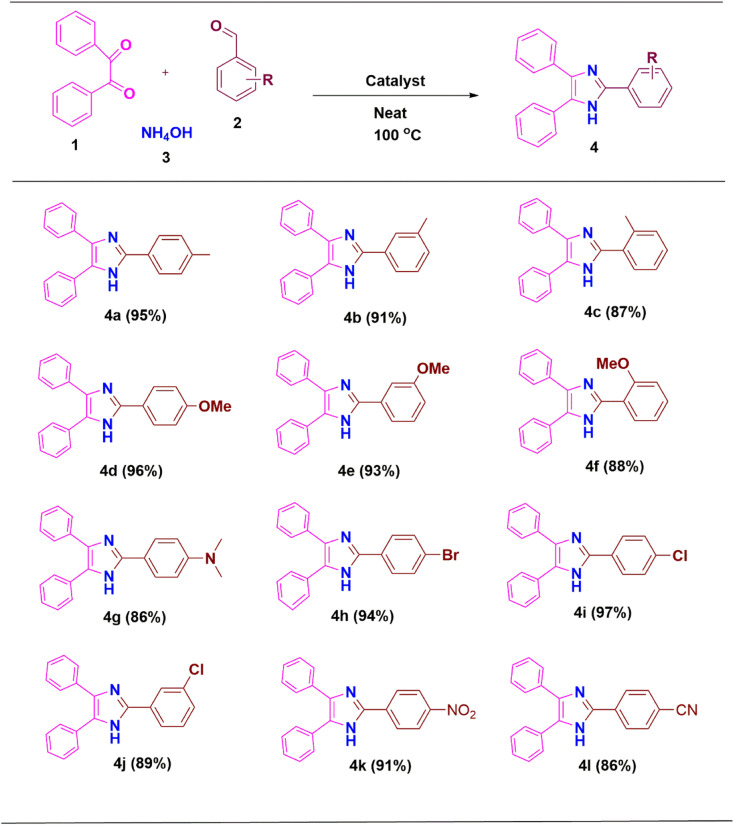
ZnO/CuI/PPy nanocatalyst catalyzed synthesis of 2,4,5-trisubstituted imidazole derivatives. ^a^Reaction conditions: benzil 1 (1 mmol), aldehyde 2 (1 mmol), ammonium acetate 3 (5 mmol), ZnO/CuI/PPy (10–30 mg) and solvent (4 mL) were stirred at 100 °C temperature.

A possible route for this reaction catalyzed by the ZnO/CuI/PPy nanocatalyst is presented in Fig. 9. The first step includes the activation of the aldehydic carbonyl oxygen by the catalyst and condensation with two molecules of ammonia, resulting in intermediate diamine A. The intermediate A further condenses with the carbonyl carbons of benzil, followed by dehydration to give the intermediate B, which results in trisubstituted imidazole C by the transfer of a proton (Scheme 1).

**Fig. 10:**
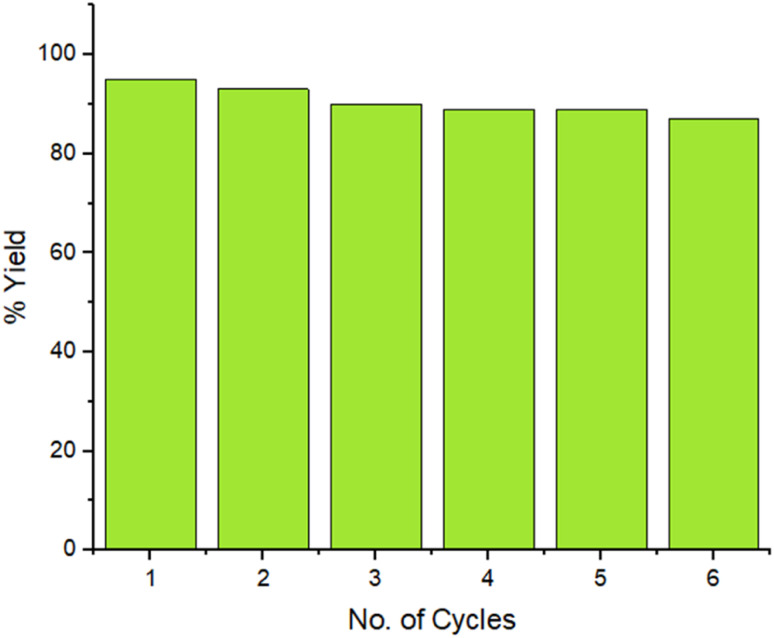
Recyclability of the ZnO/CuI/PPy nanocatalyst.

The reaction was setup on a large scale and the recyclability of the ZnO/CuI/PPy nanocatalyst was examined for six repeated cycles for benzil (1), *p*-tolualdehyde (2) and ammonium acetate (3) as a model reaction under suitable conditions, as presented in Fig. 10. The outcomes undoubtedly showed that there was no substantial loss in the catalytic efficacy of the material over six consecutive rounds which was confirmed by FESEM, TEM, and XRD (ESI Fig. S1–S3†). The ICP analysis of the filtrate was measured after the removal of the catalyst from the reaction mixture and it was found that leached concentrations for metal ion copper and zinc are 2.08 and 0.12 ppm, respectively, that are lesser than the authentic concentration value of the respective ions, according to WHO terms.^38^

The present methodology is sustainable as the values of green metrics are near the ideal values as shown in Table 2.

Table 3 shows the comparison of the ZnO/CuI/PPy nanocatalyst with various catalysts for the creation of 2,4,5-trisubstituted imidazole. It can be seen that the current catalyst has better results when compared with various other catalysts.

**Table 3:** Comparison of catalytic efficiency of the ZnO/CuI/PPy catalyst with that of previously reported catalysts

S. No.	Catalyst	Reaction conditions	Temperature	Time	Yield (%)	Ref.
1	InCl_3_·3H_2_O	Methanol	r.t.	8.3 h	82	40
2	PTSA	Ethanol	80 °C	1 h	84	41
3	I_2_	Ethanol	75 °C	15 min	99	42
4	Yb(OPf)_3_	Fluorous solvents	80 °C	6 h	97	43
5	CS-SO_3_H	Solvent-free	110 °C	90 min	93	44
6	[HbIm]BF_4_	Solvent-free	100 °C	60 min	95	45
7	Fe_3_O_4_/SiO_2_/urea	Ethanol	80 °C	50 min	87	46
8	HNO_3_@nanoSiO_2_	Solvent-free	100 °C	2.15 h	92	47
9	ZnO/CuI/PPy	Neat	100 °C	40 min	95	This work

## 3 Experimental section

### 3.1 Preparation of ZnO nanoparticles

The ZnO nanoparticles were synthesized by the co-precipitation method using ZnCl_2_ as a precursor. Briefly, 3 g of ZnCl_2_ was added to 100 mL of distilled water with continuous stirring at 70 °C. After 5 min, 8 mL of NH_4_OH was added to the solution, and stirred for 2 hours at 70 °C. Afterwards, the solid was recovered by centrifugation, washed with distilled water to remove impurities, and was dried at 60 °C for 24 hours. Finally, the material was calcined at 300 °C for 2 hours to obtain ZnO nanoparticles.^48^

### 3.2 Preparation of the ZnO/CuI/PPy nanocatalyst

Initially, 0.2 g of ZnO, 1 mol potassium iodide, 0.5 mol CuSO_4_·5H_2_O and 0.3 mL of pyrrole monomer were added to 50 mL water and the resultant solution was continuously stirred for 12 hours at ambient temperature. The obtained product was separated by centrifugation and thoroughly washed with distilled water and ethanol numerous times to eliminate impurities, and then dried overnight in an oven at 50 °C for 12 hours.

### 3.3 Procedure for the synthesis of 2,4,5-trisubstituted imidazoles

A combination of benzil (1 mmol), aldehyde (1 mmol), ammonium acetate (5 mmol) and ZnO/CuI/PPy nanocatalyst (20 mg) was stirred at 100 °C in an oil bath. The progress of the reaction was checked by TLC. After the reaction was over, the catalyst was removed by filtration. The organic layer was concentrated under reduced pressure, and the product was purified by column chromatography to give the pure 2,4,5-trisubstituted imidazole derivatives.

## 4 Conclusion

In summary, we developed a ZnO/CuI/PPy nanocatalyst as an effective and robust catalyst for the preparation of 2,4,5-trisubstituted imidazole from benzil, aldehyde, and ammonium acetate under sustainable conditions. The material was reused for six consecutive cycles with no significant loss in its catalytic activity. The current methodology is beneficial due to the easy protocol for catalyst preparation, neat conditions without additives, excellent yields and ideal values of green metrics.

### 4.1 Spectral data

#### 4.1.1 2-(4-Methylphenyl)-4,5-diphenyl-1*H*-imidazole (4a)

White solid; yield: 95%; ^1^H NMR (400 MHz, DMSO-*d*_6_) 12.57 (s, 1H), 7.97–7.95 (d, *J* = 8.25 Hz, 2H), 7.52–7.49 (d, *J* = 7.15 Hz, 4H), 7.35–7.24 (m, 8H), 2.31 (s, 3H). ^13^C NMR (100 MHz, DMSO-*d*_6_) *δ* 146.24, 138.23, 129.78, 128.92, 128.23, 127.59, 125.74, 21.42; anal. calcd for C_22_H_20_N_2_: C, 84.58; H, 6.45; N, 8.97; found C, 84.55; H, 6.43; N, 8.95.

#### 4.1.2 2-(3-Methylphenyl)-4,5-diphenyl-1*H*-imidazole (4b)

White solid; yield: 91%; ^1^H NMR (400 MHz, DMSO-*d*_6_) 12.62 (s, 1H), 7.89 (s, 1H), 7.85–7.83 (d, *J* = 7.83 Hz, 2H), 7.49–7.47 (d, *J* = 7.28 Hz, 4H), 7.34–7.22 (m, 6H), 7.16–7.14 (d, *J* = 7.83 Hz, 1H), 2.34 (s, 3H). ^13^C NMR (100 MHz, DMSO-*d*_6_) *δ* 146.16, 138.36, 130.75, 129.48, 129.14, 126.27, 122.91, 21.63; anal. calcd for C_22_H_20_N_2_: C, 84.58; H, 6.45; N, 8.97; found C, 84.61; H, 6.46; N, 8.98.

#### 4.1.3 2-(2-Methylphenyl)-4,5-diphenyl-1*H*-imidazole (4c)

White solid; yield: 87%; ^1^H NMR (400 MHz, DMSO-*d*_6_) 12.66 (s, 1H), 7.82–7.81 (d, *J* = 5.77 Hz, 4H), 7.65 (s, 4H), 7.48 (s, 4H), 7.21 (s, 2H), 2.49 (s, 3H). ^13^C NMR (100 MHz, DMSO-*d*_6_) *δ* 136.31, 135.39, 132.14, 130.96, 129.49, 128.86, 128.53, 128.07, 127.57, 127.22, 126.68, 125.69, 20.81; anal. calcd for C_22_H_20_N_2_: C, 84.58; H, 6.45; N, 8.97; found C, 84.56; H, 6.45; N, 8.96.

#### 4.1.4 2-(4-Methoxyphenyl)-4,5-diphenyl-1*H*-imidazole (4d)

White solid; yield: 96%; ^1^H NMR (400 MHz, DMSO-*d*_6_) 12.53 (s, 1H), 8.02–7.99 (d, *J* = 8.79 Hz, 2H), 7.51–7.48 (d, *J* = 7.15 Hz, 4H) 7.34–7.25 (m, 6H), 7.03–7.00 (d, *J* = 8.93 Hz, 2H), 3.77 (s, 3H). ^13^C NMR (100 MHz, DMSO-*d*_6_) *δ* 159.97, 146.18, 128.92, 128.24, 127.26, 123.65, 114.62, 55.73; anal. calcd for C_22_H_20_N_2_O: C, 80.46; H, 6.14; N, 8.53; found C, 80.45; H, 6.16; N, 8.53.

#### 4.1.5 2-(3-Methoxyphenyl)-4,5-diphenyl-1*H*-imidazole (4e)

White solid; yield: 93%; ^1^H NMR (400 MHz, DMSO-*d*_6_) 12.68 (s, 1H), 7.68–7.65 (d, *J* = 12.23 Hz, 2H), 7.52–7.50 (d, *J* = 12.23 Hz, 4H), 7.37–7.27 (m, 7H), 6.92–6.90 (d, *J* = 10.03 Hz, 1H), 3.80 (s, 3H). ^13^C NMR (100 MHz, DMSO-*d*_6_) *δ* 160.09, 145.91, 132.18, 130.36, 128.97, 127.44, 118.17, 114.76, 110.71, 55.73; anal. calcd for C_22_H_20_N_2_O: C, 80.46; H, 6.14; N, 8.53; found C, 80.49; H, 6.15; N, 8.55.

#### 4.1.6 2-(2-Methoxyphenyl)-4,5-diphenyl-1*H*-imidazole (4f)

White solid; yield: 88%; ^1^H NMR (400 MHz, DMSO-*d*_6_) 11.91 (s, 1H), 8.01–7.99 (d, *J* = 6.05 Hz, 1H), 7.47–7.45 (d, *J* = 7.42 Hz, 4H), 7.37–7.25 (m, 7H), 7.14–7.01 (m, 2H), 3.88 (s, 3H). ^13^C NMR (100 MHz, DMSO-*d*_6_) *δ* 156.54, 143.67, 130.34, 129.40, 128.90, 127.38, 121.12, 112.12, 56.08; anal. calcd for C_22_H_20_N_2_O: C, 80.46; H, 6.14; N, 8.53; found C, 80.43; H, 6.16; N, 8.57.

#### 4.1.7 2-(*N*,*N*-Dimethylaminophenyl)-4,5-diphenyl-1*H*-imidazole (4g)

White solid; yield: 86%; ^1^H NMR (400 MHz, DMSO-*d*_6_) 7.95–7.93 (d, *J* = 8.52 Hz, 2H), 7.55–7.53 (d, *J* = 8.11 Hz, 4H), 7.32–7.28 (t, *J* = 8.11 Hz, 4H), 7.25–7.21 (t, *J* = 7.97 Hz, 2H) 6.74–6.72 (d, *J* = 8.52 Hz, 2H), 3.00 (s, 6H). ^13^C NMR (100 MHz, DMSO-*d*_6_) *δ* 155.34, 151.91, 138.26, 133.18, 132.91, 131.79, 131.67, 123.15, 116.77; anal. calcd for C_23_H_23_N_3_: C, 80.90; H, 6.79; N, 12.31; found C, 80.92; H, 6.82; N, 12.33.

#### 4.1.8 2-(4-Bromophenyl)-4,5-diphenyl-1*H*-imidazole (4h)

White solid; yield: 94%; ^1^H NMR (400 MHz, DMSO-*d*_6_) 8.03 (s, 2H) 7.65–7.33 (m, 12H). ^13^C NMR (100 MHz, DMSO-*d*_6_) *δ* 145.01, 133.35, 132.25, 129.93, 128.99, 128.33, 127.70, 122.07; anal. calcd for C_21_H_17_BrN_2_: C, 66.85; H, 4.54; N, 7.43; found C, 66.81; H, 4.56; N, 7.44.

#### 4.1.9 2-(4-Chlorophenyl)-4,5-diphenyl-1*H*-imidazole (4i)

White solid; yield: 97%; ^1^H NMR (400 MHz, DMSO-*d*_6_) 8.09–8.07 (d, *J* = 8.66 Hz, 2H), 7.53–7.48 (t, *J* = 8.79 Hz, 7H) 7.35–7.32 (t, *J* = 7.01 Hz 4H), 7.28–7.25 (m, 1H). ^13^C NMR (100 MHz, DMSO-*d*_6_) *δ* 144.85, 133.67, 132.97, 129.36, 129.02, 128.36, 127.98, 127.58; anal. calcd for C_21_H_17_ClN_2_: C, 75.78; H, 5.15; N, 8.42; found C, 75.74; H, 5.13; N, 8.46.

#### 4.1.10 2-(3-Chlorophenyl)-4,5-diphenyl-1*H*-imidazole (4j)

White solid; yield: 89%; ^1^H NMR (400 MHz, DMSO-*d*_6_) 12.80 (s, 1H), 8.13 (s, 2H), 8.03–8.01 (d, *J* = 7.83 Hz, 1H), 7.51–7.45 (m, 7H) 7.40–7.28 (m, 4H). ^13^C NMR (100 MHz, DMSO-*d*_6_) *δ* 144.52, 134.12, 132.81, 131.22, 128.98, 128.44, 125.19, 124.22; anal. calcd for C_21_H_17_ClN_2_: C, 75.78; H, 5.15; N, 8.42; found C, 75.77; H, 5.18; N, 8.44.

#### 4.1.11 2-(4-Nitrophenyl)-4,5-diphenyl-1*H*-imidazole (4k)

Yellow solid; yield: 91%; ^1^H NMR (400 MHz, DMSO-*d*_6_) 8.34–8.28 (m, 2H), 7.91–7.89 (d, *J* = 7.56 Hz, 4H), 7.76–7.72 (t, *J* = 7.28 Hz, 4H), 7.59–7.50 (m, 4H). ^13^C NMR (100 MHz, DMSO-*d*_6_) *δ* 145.01, 133.35, 132.25, 129.93, 128.99, 128.33, 127.70, 122.07; anal. calcd for C_21_H_15_N_3_O_2_: C, 73.89; H, 4.43; N, 12.31; found C, 73.89; H, 4.44; N, 12.30.

#### 4.1.12 2-(4-Cyanophenyl)-4,5-diphenyl-1*H*-imidazole (4l)

White solid; yield: 86%; ^1^H NMR (400 MHz, DMSO-*d*_6_) 13.02 (s, 1H), 8.27–8.25 (d, *J* = 7.83 Hz, 1H), 8.06–8.05 (d, *J* = 5.08 Hz, 1H), 7.92–7.87 (m, 2H), 7.64–7.62 (d, *J* = 7.42 Hz, 3H), 7.51–7.38 (m, 7H). ^13^C NMR (100 MHz, DMSO-*d*_6_) *δ* 163.98, 134.02, 131.71, 131.13, 130.18, 127.91, 126.32, 122.15, 117.88, 115.41, 115.02; anal. calcd for C_22_H_15_N_3_: C, 82.22; H, 4.70; N, 13.08; found C, 82.23; H, 4.68; N, 13.09.

## Author contributions

S. K., N., G. R., S. H., and R. C. designed the schemes. S. K. and N. performed the experiments. S. K., and G. R. evaluated the data and prepared the figures and tables. S. K., N., G. R., S. .H., and R. C. revised and reviewed the manuscript.

## Conflicts of interest

The authors declare no competing financial interest.

## Supplementary Material

NA-005-D3NA00077J-s001
