# Supplementary material for: Exploring the untapped catalytic application of a ZnO/CuI/PPy nanocomposite for the green synthesis of biologically active 2,4,5-trisubstituted imidazole scaffolds
Source: Nanoscale Adv. 2023 Mar 20;5(8):2352–60. doi: 10.1039/d3na00077j (PMC10089100; doi:10.1039/d3na00077j)
Supplement: NA-005-D3NA00077J-s001 [file NA-005-D3NA00077J-s001.pdf]

Electronic Supplementary Material (ESI) for Nanoscale Advances.
This journal is © The Royal Society of Chemistry 2023

Exploring the untapped catalytic application of ZnO/CuI/PPy nanocomposite for the green synthesis of biologically active 2,4,5-trisubstituted imidazole scaffolds

Sahil Kohli,[1,2] Nisha, [1] *Garima Rathee,*[1] *Sunita Hooda*[2,a*] *and Ramesh Chandra*[1,3,4,b*]

1- Drug Discovery & Development Laboratory, Department of Chemistry, University of Delhi, Delhi-110007, India.

2- Department of Chemistry, Acharya Narendra Dev College, University of Delhi, Delhi-110019, India.

3- Dr. B.R. Ambedkar Center for Biomedical Research (ACBR), University of Delhi, Delhi-110007, India.

4- Institute of Nanomedical Science (INMS), University of Delhi, Delhi-110007, India.

***Correspondence:**

(a) Dr. Sunita Hooda, *Department of Chemistry, Acharya Narendra Dev College, University of Delhi, Delhi-110019, India.* E-mail: hooda_sunita@hotmail.com

(b) Prof. Ramesh Chandra, Drug Discovery and Development Laboratory, Department of Chemistry, University of Delhi, Delhi-110007, India. E-mail: rameshchandragroup@gmail.com.

ELECTRONIC SUPPLEMENTARY INFORMATION (ESI)

Electronic Supplementary Information (ESI) includes FESEM, TEM and XRD of recycled ZnO/CuI/PPy nanocatalyst; Green chemistry metric calculations, ^{1}H NMR and ^{13}C NMR spectra of compounds.

Total No of Pages: 17, Total No of Tables: 0, Total number of Figures: 27.

Table of contents

**Serial number**	**Content**	**Page number**
1.	General Remarks	3
2.	Characterization of Recycled catalyst	4
4.	Calculation of Green chemistry metrics	5
8.	^{1}H and ^{13}C spectra of compounds	6-17

General Remarks

Chemicals and solvents were purchased from Sigma Adrich, Alfa-Aesar and Merck India Pvt. ^{1}H and ^{13}C spectra were recorded on a Jeol Spectrospin spectrometer at 400 MHz and 100 MHz respectively by keeping TMS as internal standard. CHNS Analyser was recorded on model Vario Micro Cube at USIC (University Science Instrument Centre), University of Delhi, Delhi, India. The X-ray diffractometer (Model No. D8 DISCOVER) at 2θ range of 2–90° with Cu Kα radiation. Chemical shift values were recorded in terms of δ and coupling constants (J) are in hertz (Hz). FTIR spectra were obtained on IRAffinity-1S Fourier Transform Infrared Spectrophotometer. FESEM measurement was performed on Zeiss GeminiSEM 500 at USIC, University of Delhi. Transmission electron microscopy (TEM) was obtained on a TECNAI G20 HR-TEM 200kV at SAIF (sophisticated analytical instrumentation facility), AIIMS, New Delhi, India. The elemental composition and electronic structure analysis were obtained from X-ray photoelectron spectra (XPS) of PHI 5000 Versa Probe III instrument at Institute Instrumentation Centre, Indian Institute of Technology, Roorkee – 247 667 (Uttarakhand), India. ICP-MS was measured on Agilent ICP-MS 7900 with UHMI at the ICP-MS Lab, (CRF Adopted Facility), IIT Delhi, INDIA.

Characterization of Recycled ZnO/CuI/PPy nanocatalyst

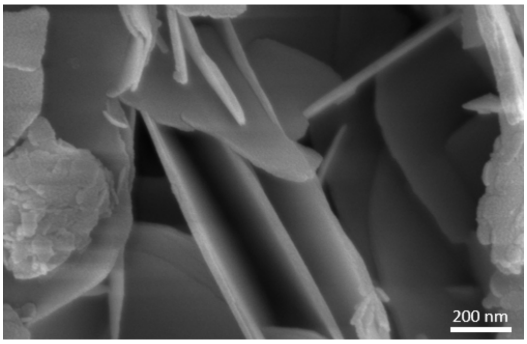

Figure S1: FESEM of recycled nanocatalyst

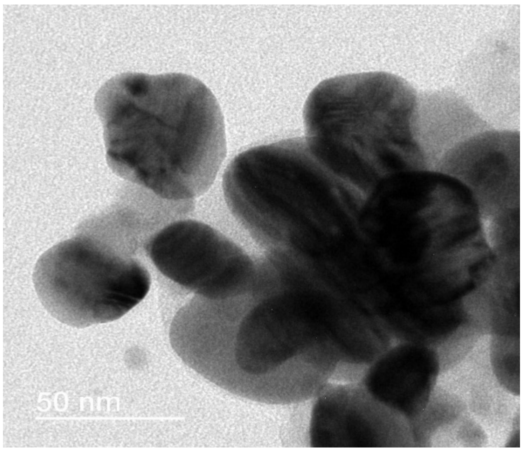

Figure S2: TEM of recycled nanocatalyst

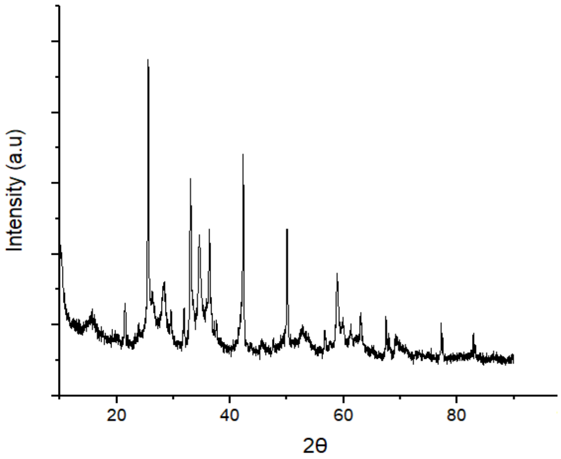

Figure S3: XRD of recycled nanocatalyst

Calculation of Green chemistry metrics

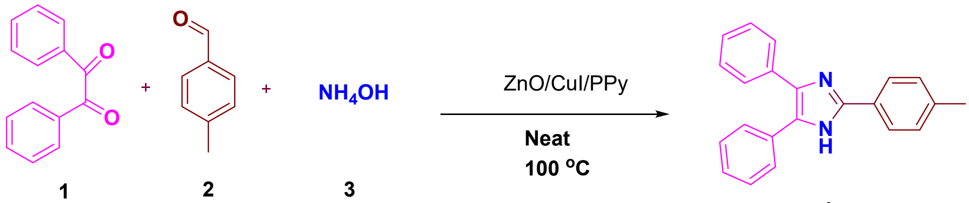

1 2 3 4a

	1	2	3	4a
**FW**	210.23 g/mol	120.15 g/mol	77.0825 g/mol	310.40 g/mol
**mmol**	**1**	**1**	**5**	**0.95**
**amount**	**210 mg**	**120 mg**	**385 mg**	**295 mg**

E-factor:

The ideal value of E-factor is zero.

E-factor = [total mass of raw materials - the total mass of product]/ mass of product.

E-factor of 4a = [(210 + 120+385)-295]/295

= 1.42.

Process mass intensity (PMI):

PMI= ∑ (mass of stoichiometric reactants)/[mass of product]

= (210+ 120+385)/ 295

= 2.42

Reaction mass efficiency (RME):

RME = [mass of product /∑ (mass of stoichiometric reactants)] × 100

= [295/ (210 + 120+ 385)] × 100

= 41.26%

Carbon efficiency (CE):

CE= [Amount of carbon in product/ Total carbon present in reactants] x 100

= [no. of moles of product x no. of carbons in product / (moles of a x carbons in 1i + moles of 2 carbons in 2)] x 100

= [0.95 x 22 / (1 x 14 + 1 x 8)] x 100

= [20.9 / (22)] x 100

= 95%

^{1}H and ^{13}C spectra of compounds

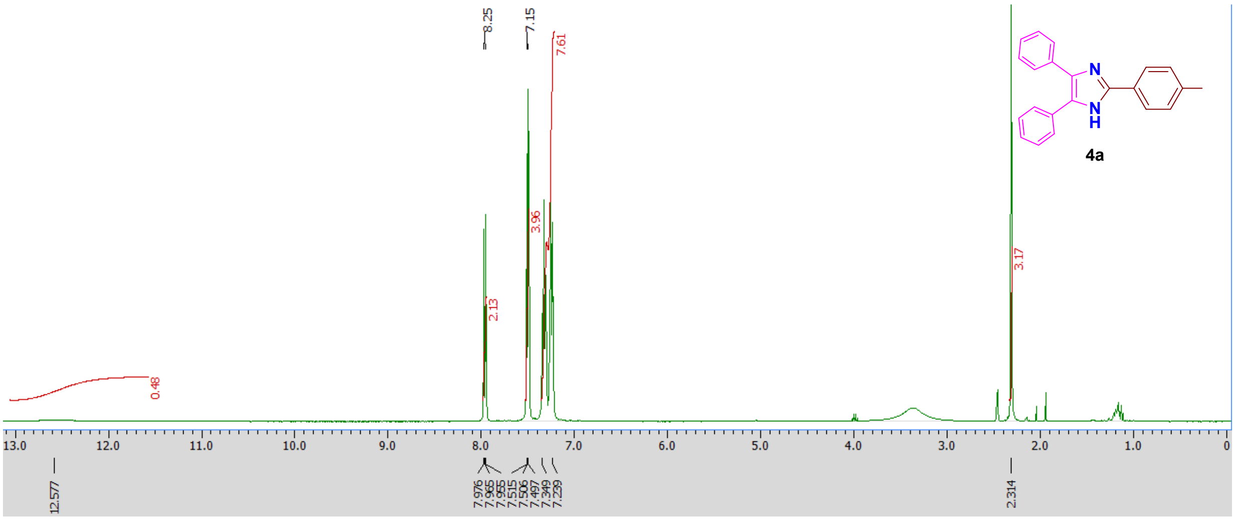

Figure S4: ^{1}H NMR of 2-(4-Methylphenyl)-4,5-diphenyl-1H-imidazole

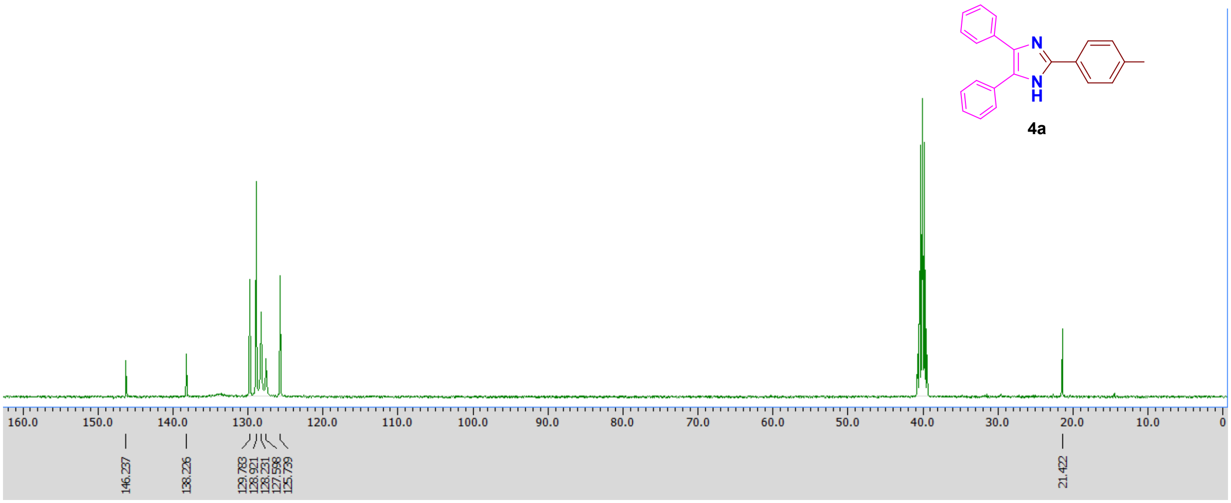

Figure S5: ^{13}C NMR of 2-(4-Methylphenyl)-4,5-diphenyl-1H-imidazole

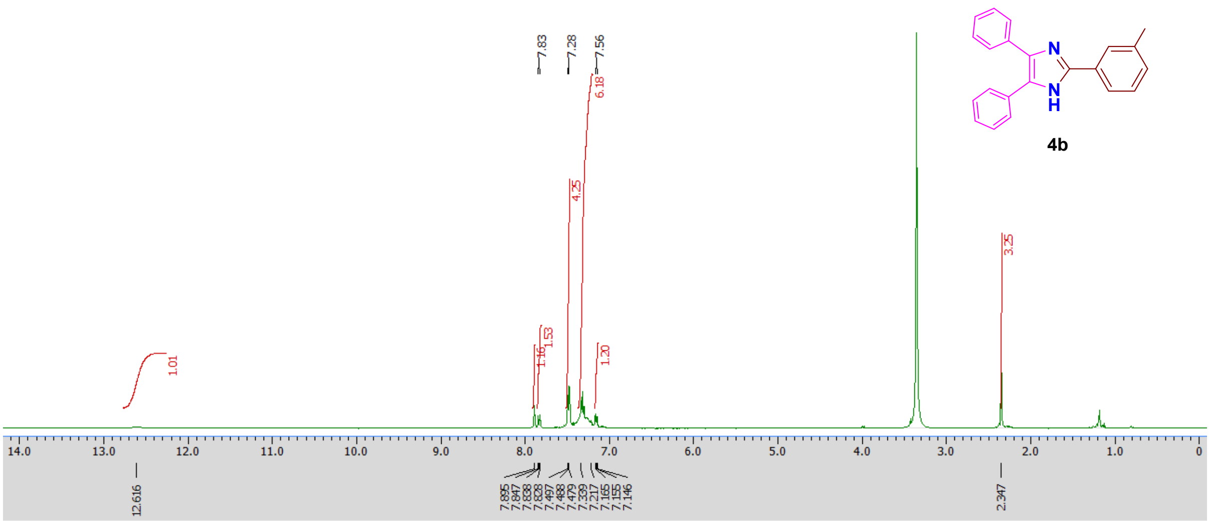

Figure S6: ^{1}H NMR of 2-(3-Methylphenyl)-4,5-diphenyl-1H-imidazole

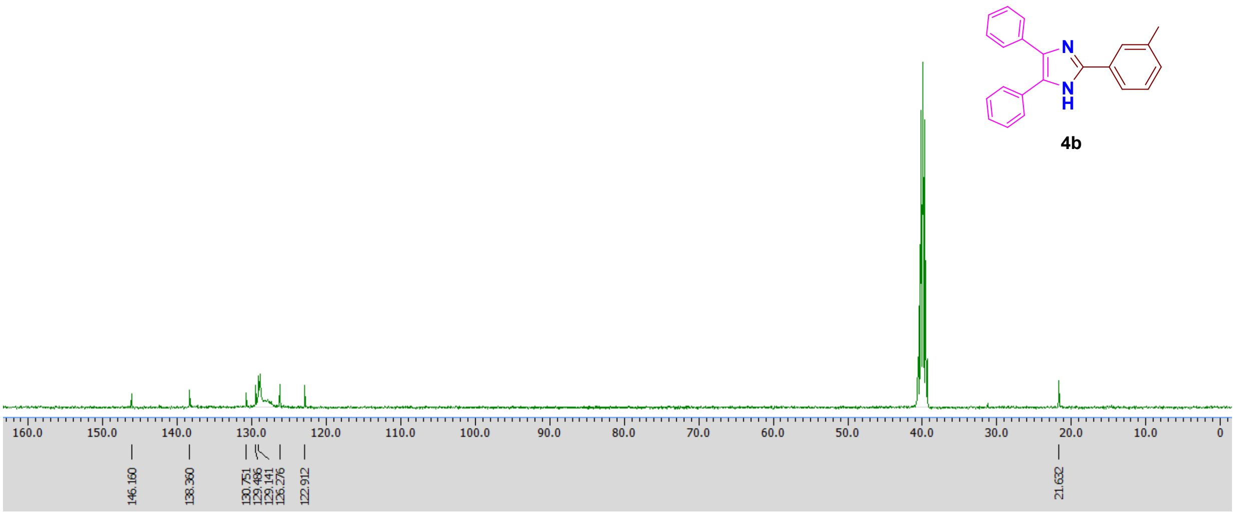

Figure S7: ^{13}C NMR of 2-(3-Methylphenyl)-4,5-diphenyl-1H-imidazole

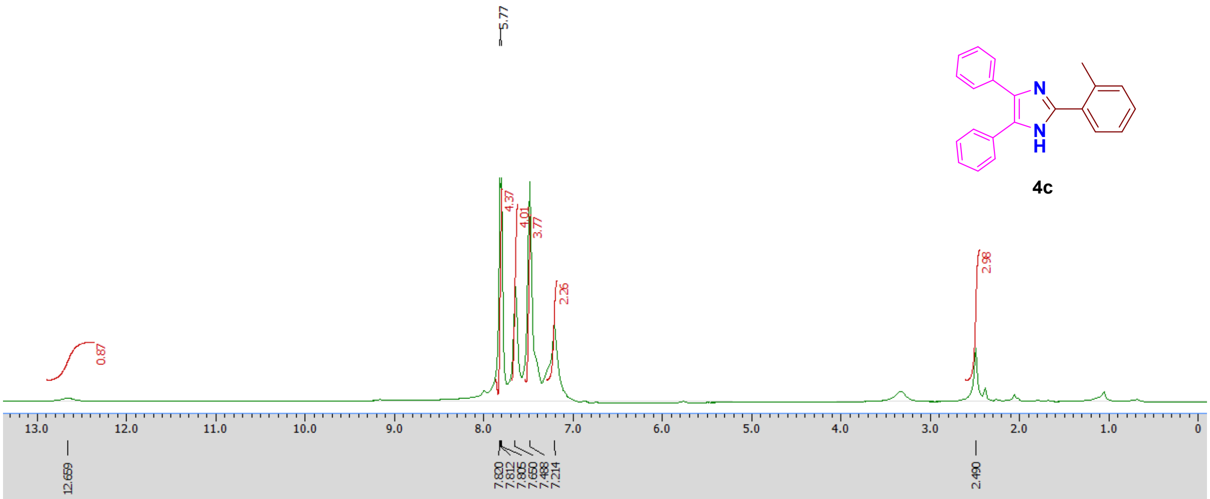

Figure S8: ^{1}H NMR of 2-(2-Methylphenyl)-4,5-diphenyl-1H-imidazole

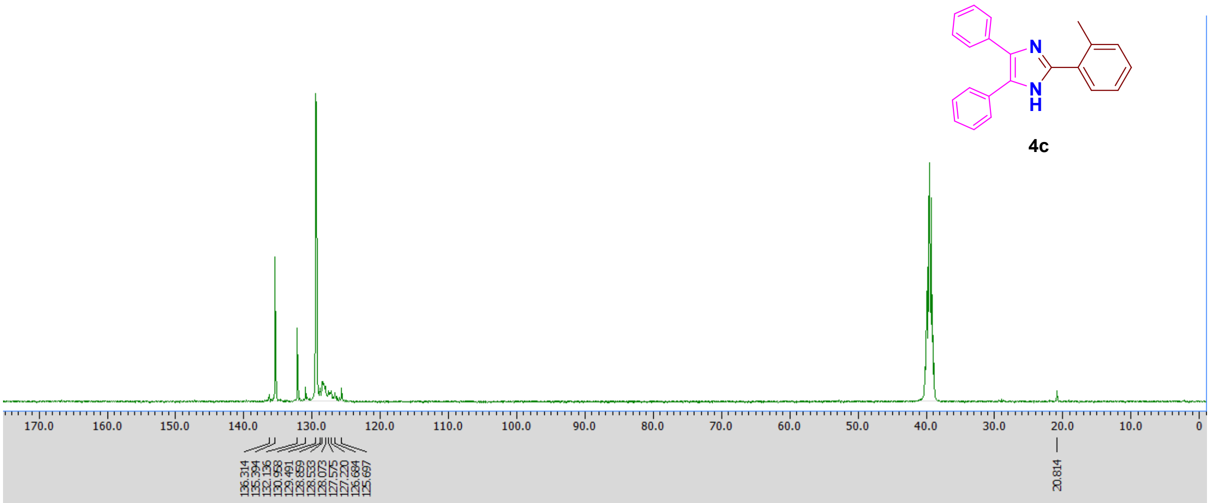

Figure S9: ^{13}C NMR of 2-(2-Methylphenyl)-4,5-diphenyl-1H-imidazole

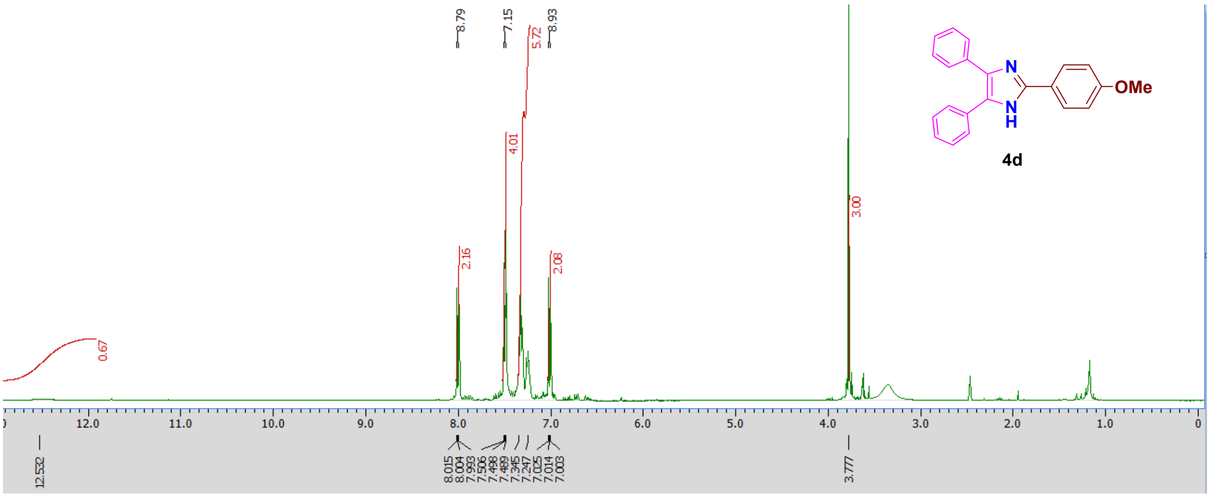

Figure S10: ^{1}H NMR of 2-(4-Methoxyphenyl)-4,5-diphenyl-1H-imidazole

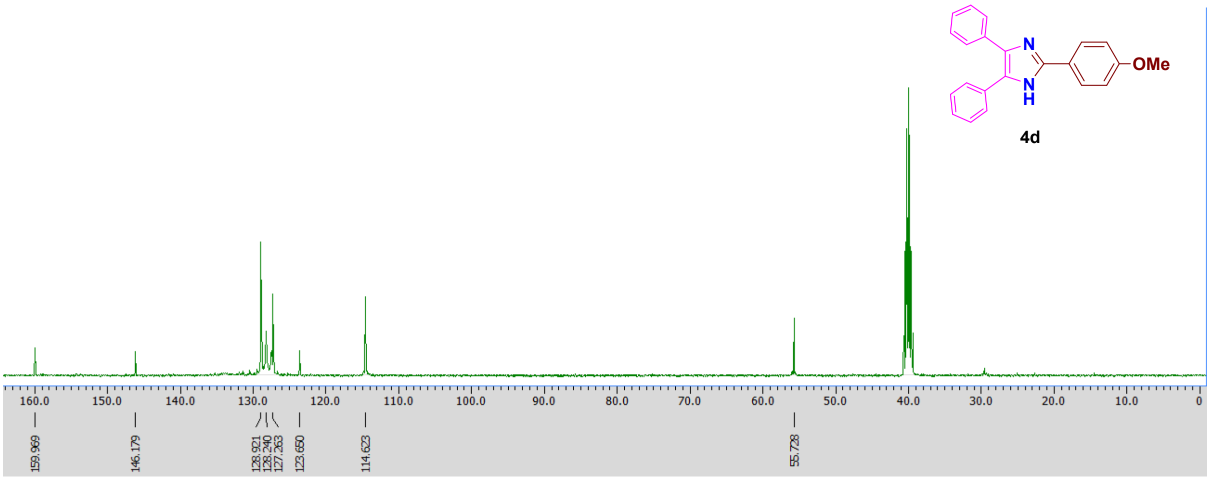

Figure S11: ^{13}C NMR of 2-(4-Methoxyphenyl)-4,5-diphenyl-1H-imidazole

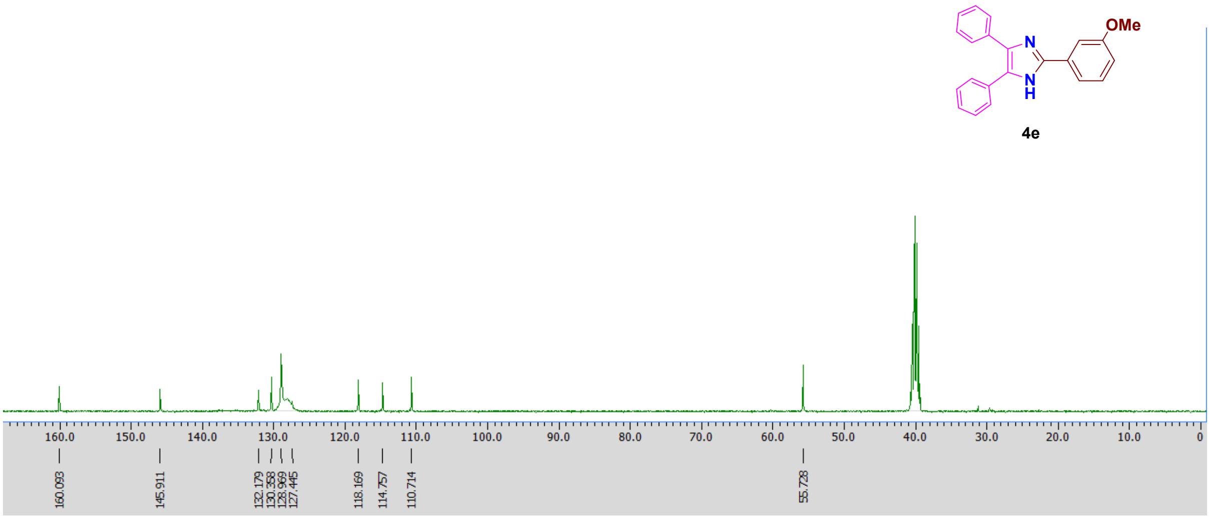

Figure S12: ^{1}H NMR of 2-(3-Methoxyphenyl)-4,5-diphenyl-1H-imidazole

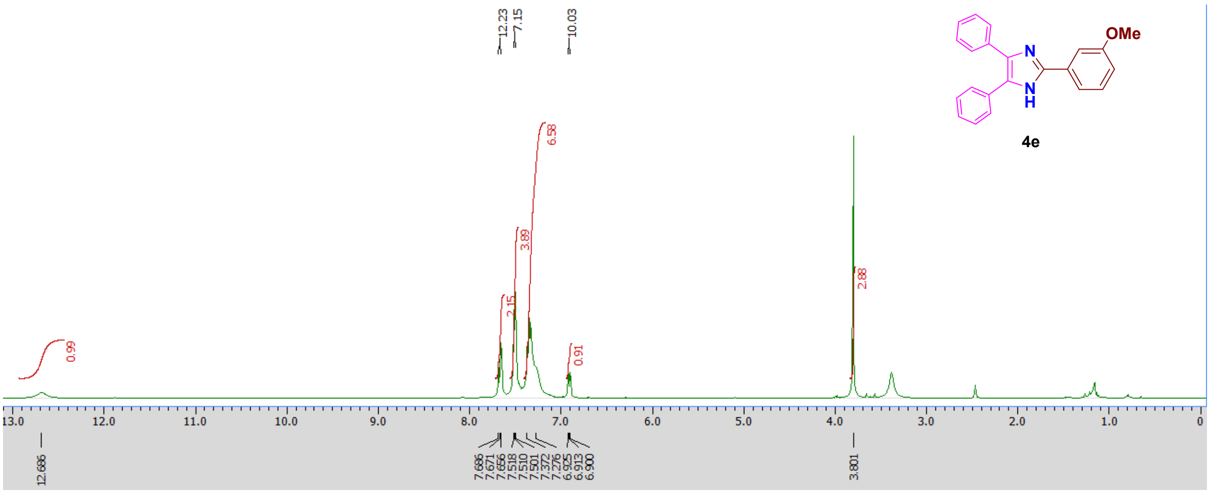

Figure S13: ^{13}C NMR of 2-(3-Methoxyphenyl)-4,5-diphenyl-1H-imidazole

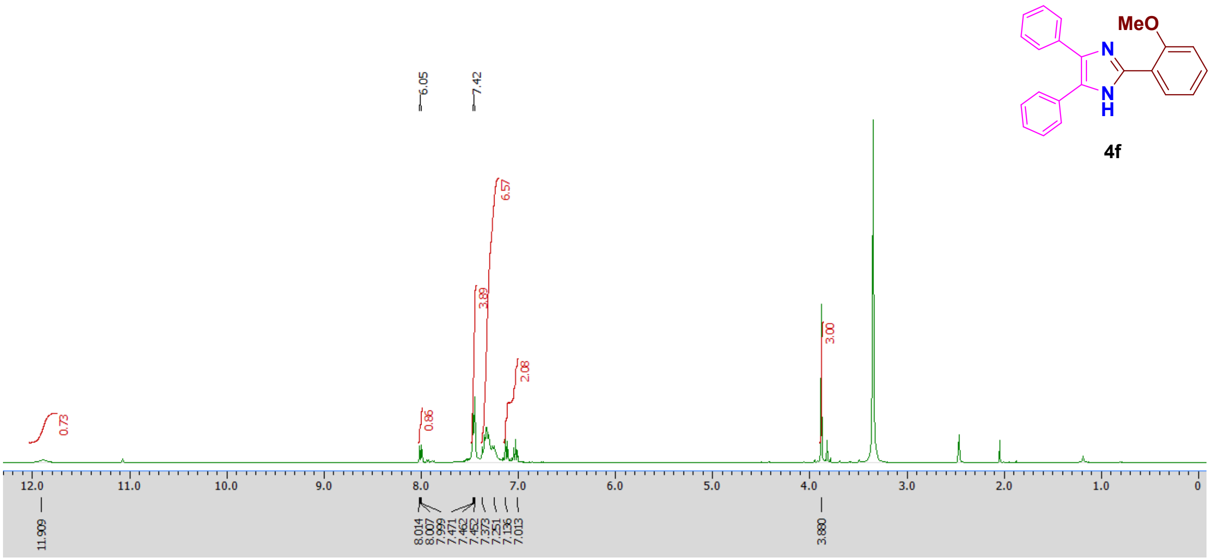

Figure S14: ^{1}H NMR of 2-(2-Methoxyphenyl)-4,5-diphenyl-1H-imidazole

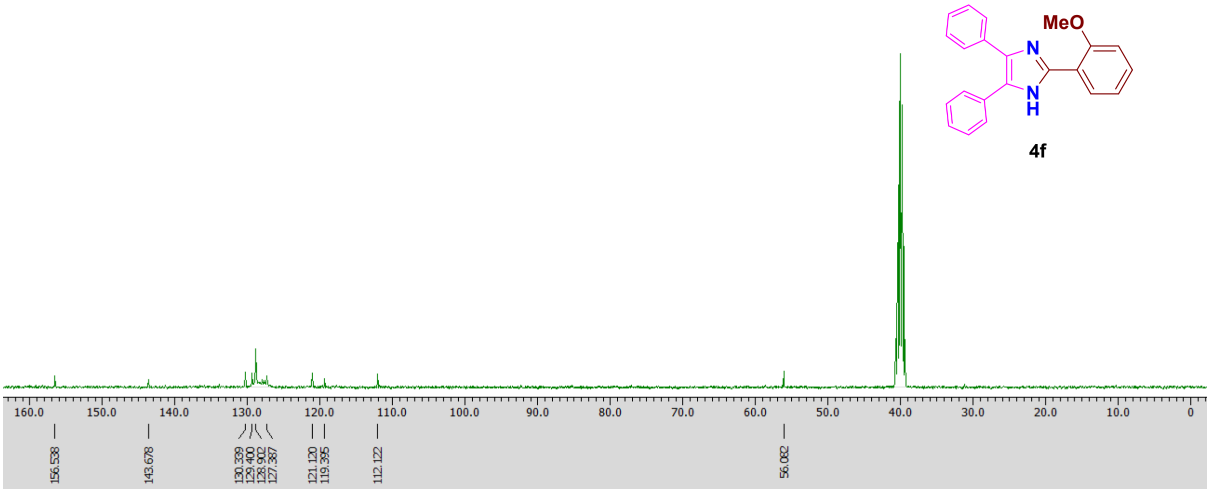

Figure S15: ^{13}C NMR of 2-(2-Methoxyphenyl)-4,5-diphenyl-1H-imidazole

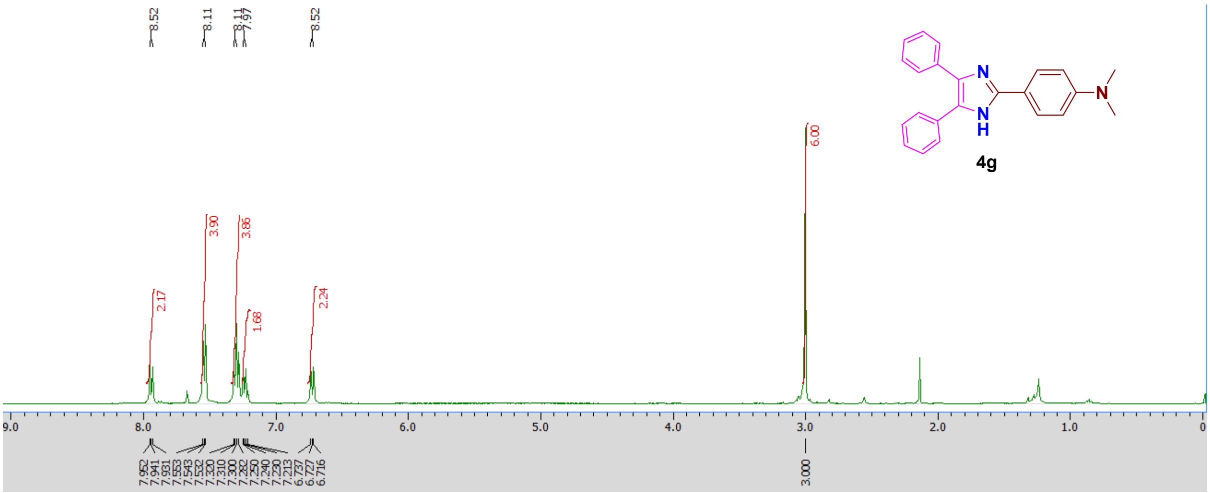

Figure S16: ^{1}H NMR of 2-(N,N-dimethylaminophenyl)-4,5-diphenyl-1H-imidazole

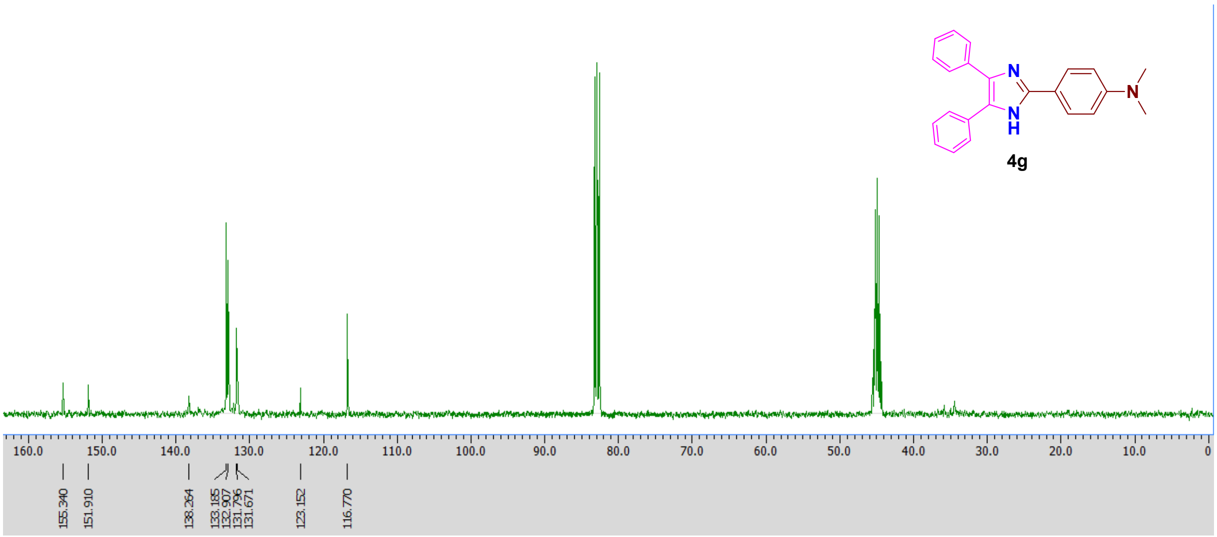

Figure S17: ^{13}C NMR of 2-(N,N-dimethylaminophenyl)-4,5-diphenyl-1H-imidazole

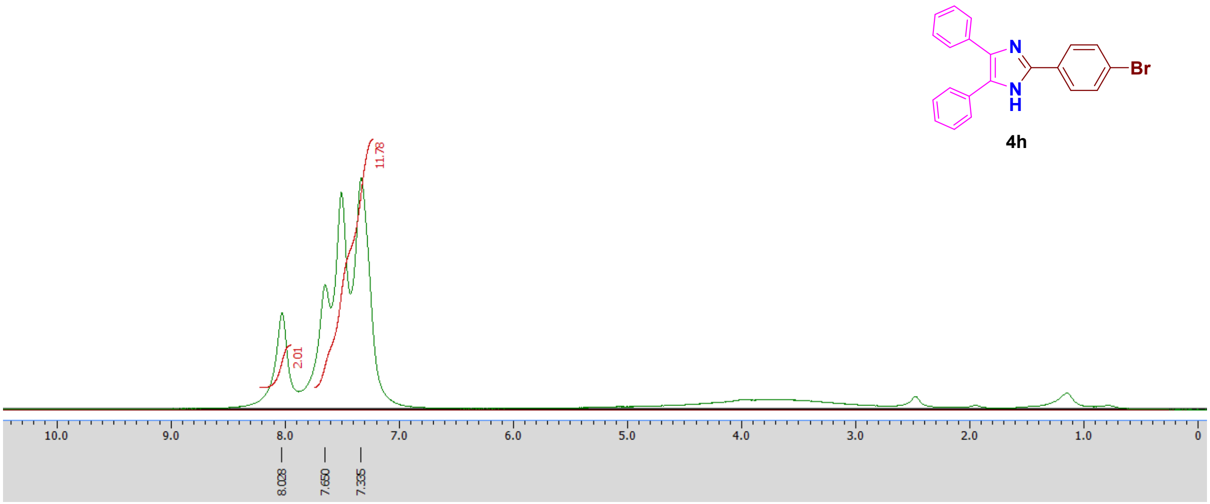

Figure S18: ^{1}H NMR of 2-(4-Bromophenyl)-4,5-diphenyl-1H-imidazole

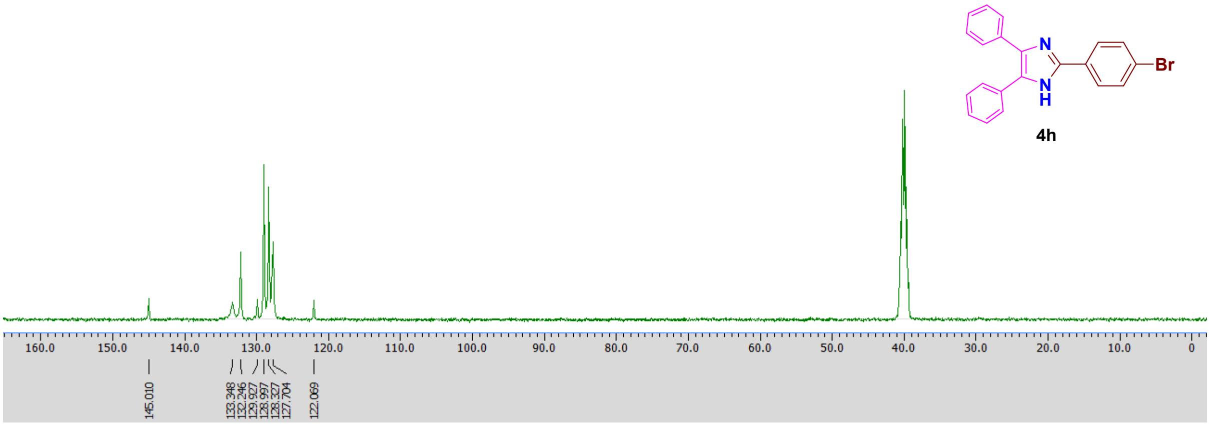

Figure S19: ^{13}C NMR of 2-(4-Bromophenyl)-4,5-diphenyl-1H-imidazole

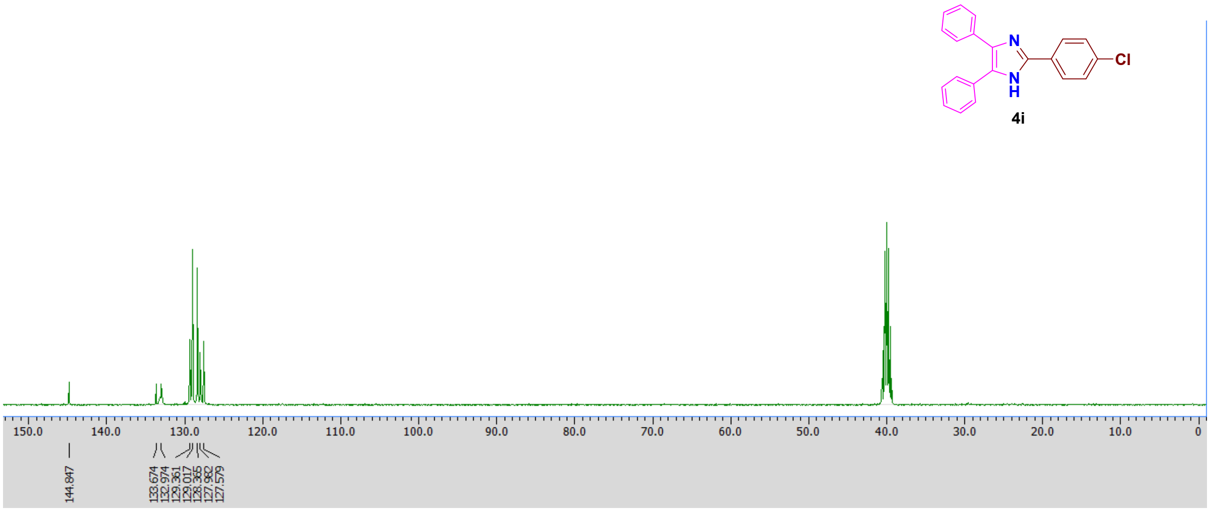

Figure S20: ^{13}C NMR of 2-(4-Chlorophenyl)-4,5-diphenyl-1H-imidazole

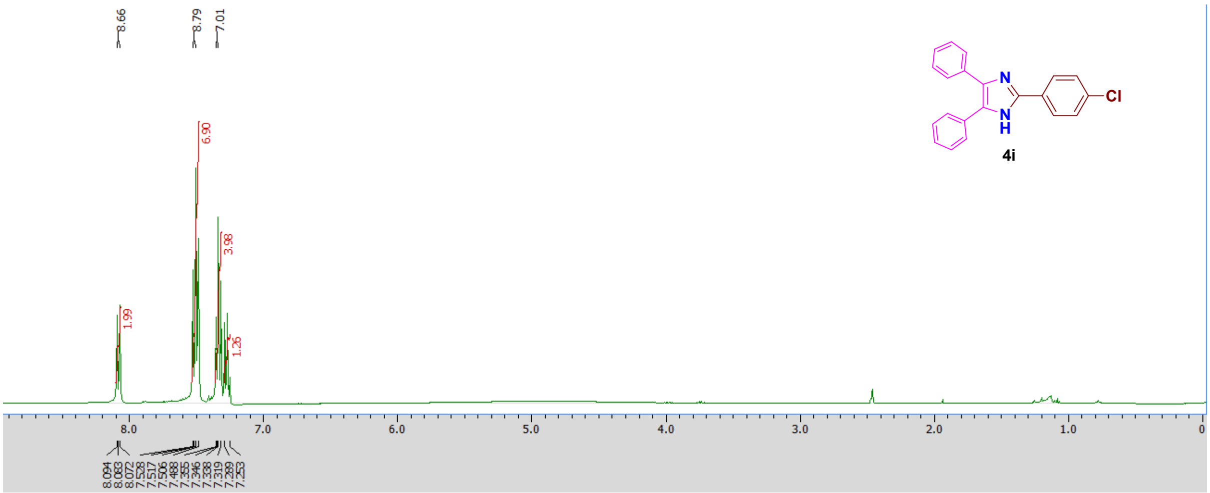

Figure S21: ^{1H} NMR of 2-(4-Chlorophenyl)-4,5-diphenyl-1H-imidazole

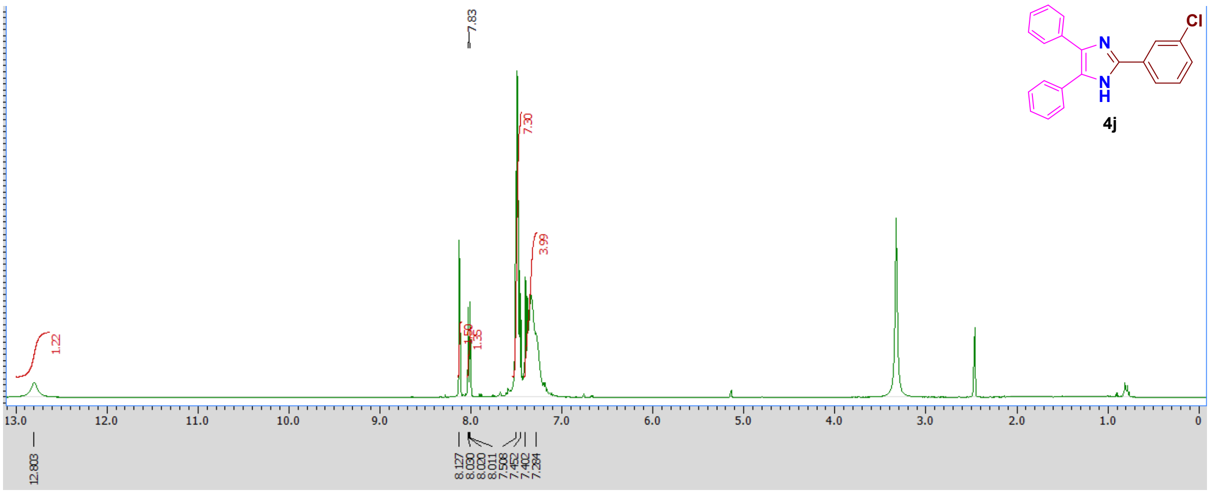

Figure S22: ^{1}H NMR of 2-(3-Chlorophenyl)-4,5-diphenyl-1H-imidazole

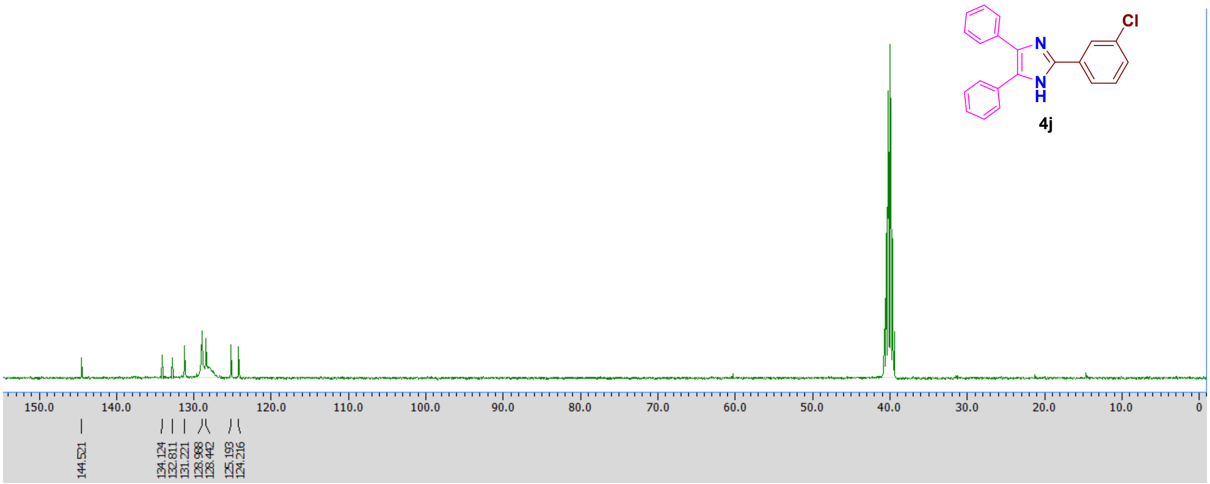

Figure S23: ^{13}C NMR of 2-(3-Chlorophenyl)-4,5-diphenyl-1H-imidazole

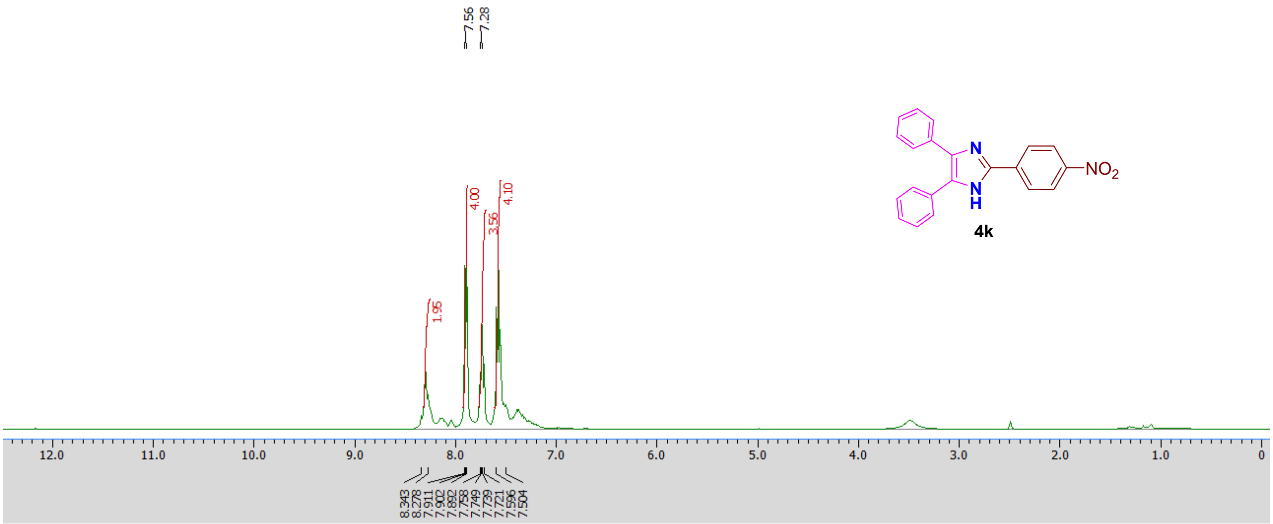

Figure S24: ^{1}H NMR of 2-(4-nitrophenyl)-4,5-diphenyl-1H-imidazole

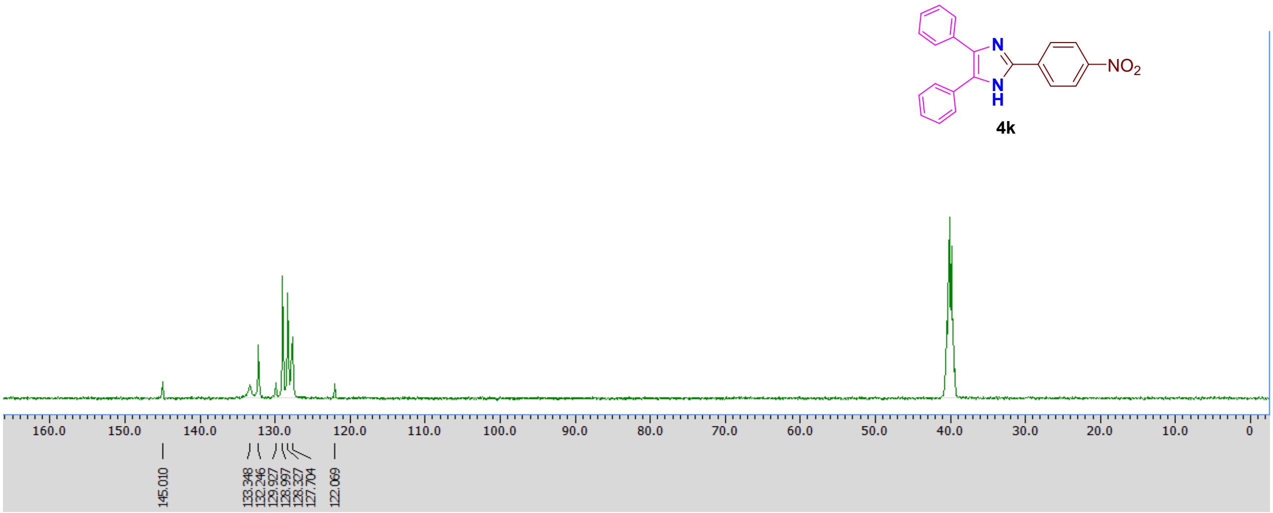

Figure S25: ^{13}C NMR of 2-(4-nitrophenyl)-4,5-diphenyl-1H-imidazole

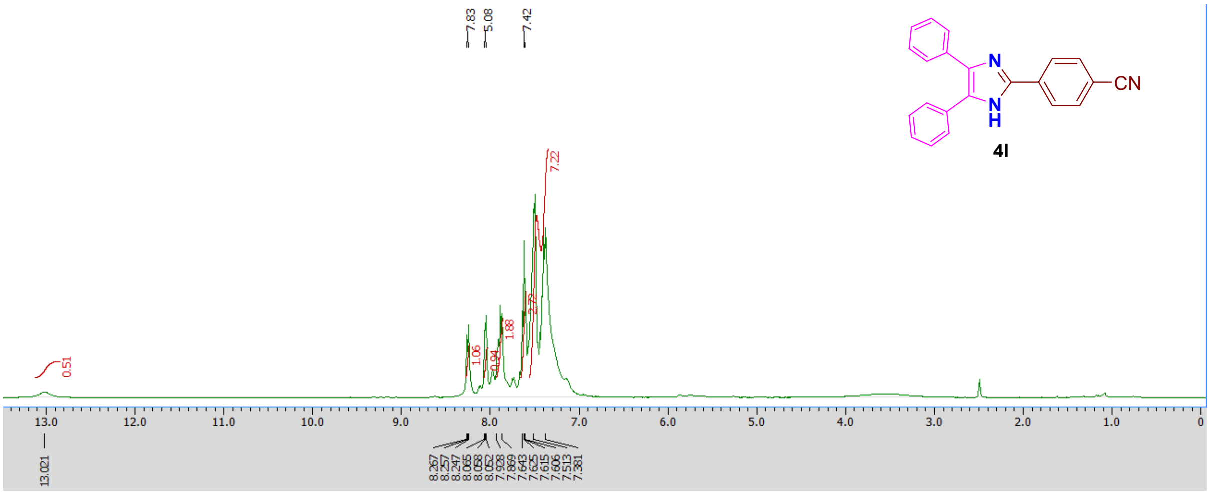

Figure S26: ^{1}H NMR of 2-(4-cyanophenyl)-4,5-diphenyl-1H-imidazole

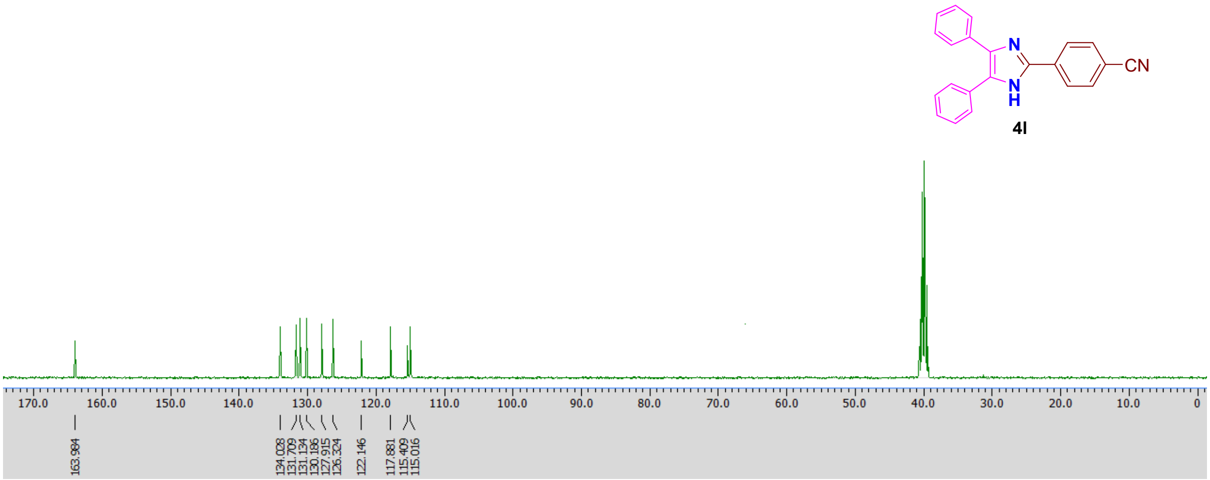

Figure S27: ^{13}C NMR of 2-(4-cyanophenyl)-4,5-diphenyl-1H-imidazole
